# Short-Term Consequences of Pediatric Anti-cancer Treatment Regarding Blood Pressure, Motor Performance, Physical Activity and Reintegration Into Sports Structures

**DOI:** 10.3389/fped.2020.00463

**Published:** 2020-08-11

**Authors:** Tina Keiser, Dominik Gaser, Christiane Peters, Renate Oberhoffer-Fritz, Sabine Kesting, Irene von Luettichau

**Affiliations:** ^1^Department of Sports Medicine and Exercise, Justus-Liebig University Gießen, Gießen, Germany; ^2^Department of Sport and Health Sciences, Institute of Preventive Pediatrics, Technical University of Munich, Munich, Germany; ^3^Department of Pediatrics and Children's Cancer Research Center, Kinderklinik München Schwabing, TUM School of Medicine, Technical University of Munich, Munich, Germany

**Keywords:** childhood cancer, cardiovascular health, motor performance, physical activity, sports, reintegration, blood pressure, arterial stiffness

## Abstract

**Background:** Cardiovascular diseases in childhood cancer survivors are known late sequelae following treatment. Arterial stiffness, pulse wave velocity (PWV) and central systolic blood pressure (cSBP) are potential predictors to assess the status of cardiovascular health. Frequent inpatient stays and reduced physical activity (PA) during treatment can lead to noticeable impairments regarding motor skills and physical performance. The present study examined parameters of cardiovascular health, motor performance and the status of integration into sports structures shortly after cessation of treatment.

**Methods:** A cross-sectional, monocentric study was conducted from April to June 2019. Participants (6–18 yrs, mixed cancer entities) during maintenance therapy and follow-up care were recruited. Peripheral and central systolic/diastolic blood pressure (pSBP, pDBP, cSBP) and PWV were assessed using the Mobil-O-Graph®. The MOON test (MOtor performance in pediatric ONcology) was used to scale motor performance. PA levels and status of integration into sports structures were assessed with a questionnaire referring to the KiGGS study. All measured data were compared to published reference values.

**Results:** Forty participants (11.3 ± 3.8 years, 50% female) were recruited 1.6 ± 1.8 years post-treatment. PSBP (z-score: 0.87 ± 0.67, *p* = 0.003), pDBP (0.83 ± 1.94, *p* = 0.033) and cSBP (≥8 years: 0.60 ± 1.29, *p* = 0.011) were significantly increased compared to reference values. PWV was also elevated, but not significantly. Motor performance was reduced in almost all motor abilities. Thirty-six percent of the examined group did not participate in physical education at school to the full extent. Only 17% reported 1 hour of daily moderate-to-vigorous PA as recommended for children and adolescents by the World Health Organization. Half of the participants were active sports club members before treatment, but one third did not resume their former membership.

**Conclusion:** Increased cardiovascular parameters and impaired motor performance shortly after cessation of treatment, physical inactivity, and low rates of integration into regular sports programs highlight the support needed. Young cancer patients should receive early support in coping with physical limitations preferably soon after diagnosis. Motor deficits could be reduced by applying targeted interventions. Furthermore, a regular sports therapy program during in- and outpatient care could increase engagement in PA to possibly counteract risk factors and improve cardiovascular health.

## Introduction

Extensive research and optimized treatment regimens resulted in an increase of the 5-year survival rate to 85% in the USA (1) and of the 15-year survival rate to 82% for patients under the age of 15 in Germany (2). However, childhood cancer is a rare disease. It contributes only around 1% to all malignant diseases in developed countries (3). Worldwide, 215,000 children under the age of 15 and 85,000 adolescents aged between 15 and 19 are diagnosed with cancer every year (4). As a consequence of the success in the treatment of childhood cancer, the importance of survival quality and prevention of late sequelae have received more attention during the last years.

Known negative long-term consequences of intensive treatment for childhood and adolescent cancer patients often include adverse effects on the cardiovascular system (5). Cardiovascular diseases are the most frequently reported causes of death in childhood cancer survivors following secondary tumors (6). Arterial stiffness, pulse wave velocity (PWV) and central systolic blood pressure (cSBP) are potential predictors for cardiovascular diseases frequently investigated in medical research to evaluate the status of a patient's cardiovascular health (7, 8).

PWV describes the velocity of the pressure wave in the aorta, which spreads from the left ventricle through the arterial vascular system during systole (9). Non-invasive investigation of the PWV, via ultrasound or oscillometric methods, provides information on the elasticity of the vascular system and enables early recognition of damages in the vessels. Thus, in order to detect potential structural modifications in the vascular system and indicators for arterial stiffness at an early stage, this subclinical parameter should be surveyed continually. Previous data indicate a positive correlation of PWV with arterial vascular stiffness (8, 10). Moreover, elevated PWV reflecting subclinical vascular damage was shown in pediatric patients after hematopoietic stem cell transplantation (11). On the contrary, another study investigated elevated blood pressure levels, but no statistically significant variation for PWV in pediatric cancer survivors compared to healthy children and adolescents (12).

In addition to the above-mentioned late sequelae, several problems already arise during treatment and often persist throughout survivorship. For instance, frequent long-term inpatient stays and reduced physical activity during treatment can lead to noticeably reduced physical performance of childhood cancer survivors and reintegration into sports structures might be affected throughout rehabilitation process (13, 14).

In healthy populations, reduced physical activity leads to negative consequences for cardiovascular health (15). Additionally, the necessary use of anthracyclines in almost 60% of applied therapy regimens in childhood cancer increases the risk of cardiovascular morbidity and mortality eight-fold compared to age-matched patients not receiving anthracyclines, indicating the importance of reducing such long-term consequences (16).

Due to a poor state of health and impaired immune function, sports options such as physical education at school, engagement in sports clubs or recreational sports are no longer feasible during therapy. Consequently, reintegration after cessation of treatment is associated with even more barriers due to disease- and treatment-related impairments (17, 18). Circumstances of anti-cancer treatment can lead to inactivity, resulting in deficits of fine and gross motor skills, reduced muscle strength, and poor physical fitness following treatment (19, 20). Especially, motor performance in pediatric bone tumor patients often remains reduced until at least 2 years after cessation of treatment (21). Impairments of physical performance have been shown to persist throughout survivorship (22), which may complicate the survivors' reintegration into both social and sports structures as well as the development of a long-term active lifestyle (23, 24).

According to a questionnaire-based study, childhood cancer survivors' reintegration rate into physical education at school is very low, especially after treatment for bone tumors (23). The lack of comprehensive offers of physical activity promotion and motor development might exacerbate motor impairments and problems of reintegration into sports structures (19).

Von Korn et al. (12) examined motor performance using the Fitnessgram® as well as peripheral blood pressure, central blood pressure and PWV using the Mobil-O-Graph® in children after treatment for childhood cancer (*n* = 92, aged 12.5 ± 4.2 years, 3.6 ± 2.8 years post-diagnosis). Their results show reduced motor performance of childhood cancer survivors compared to reference values of healthy children. However, no correlation could be drawn regarding cardiovascular parameters and motor performance (12).

The present cross-sectional study aimed at investigating various parameters of cardiovascular health, motor performance, and status of physical activity in children and adolescents shortly after cessation of anti-cancer treatment or during ongoing oral maintenance therapy. The collection of such data is of considerable importance for the early detection of health implications related to both disease and treatment. Moreover, the findings will help to support the development of preventive strategies regarding the health of children and adolescents treated for cancer. Appropriate strategies during (primary and secondary prevention) and following cancer treatment (tertiary prevention) need improvements.

## Materials and Methods

### Design

The cross-sectional, monocentric study was performed over a period of 3 months (April–June 2019) at our institution. The assessment of cardiovascular parameters using the Mobil-O-Graph® was followed by the MOON test (MOtor performance in pediatric ONcology) to evaluate motor performance. Finally, the participants completed a standardized questionnaire referring to the KiGGS study (German Health Interview and Examination Survey for Children and Adolescents) to collect data regarding their current level of physical activity and status of integration into sports structures. The Ethics Committee of the School of Medicine of the Technical University of Munich approved the study (project number 148/19 S-SR). Participation was voluntary and informed written consent was signed by each participant, as well as by his or her legal guardian. All data was collected encoded (pseudonym) and in accordance with privacy policy standards.

### Participants

**Figure 1:**
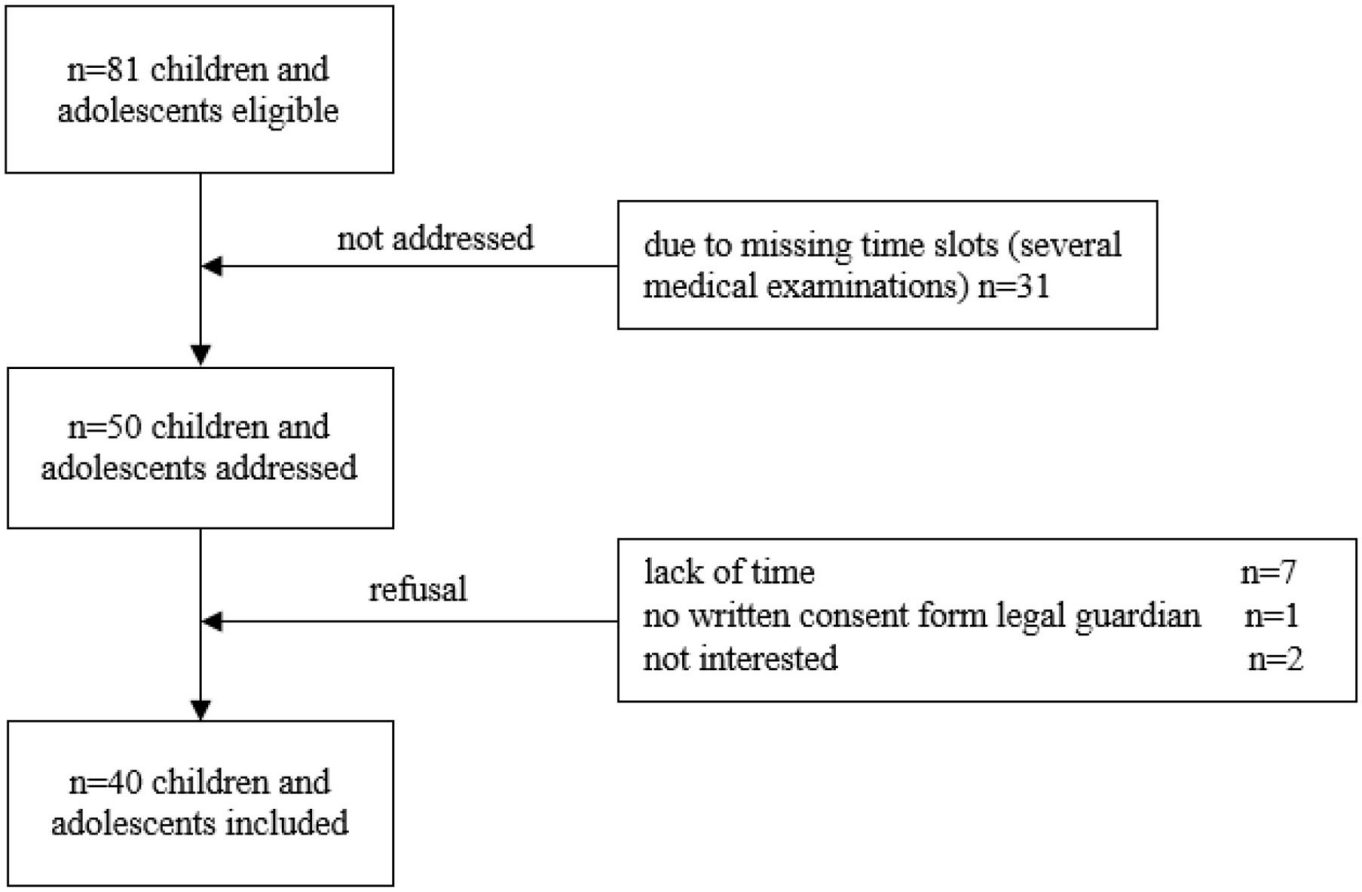
Flow chart of recruitment. Out of 81 participants eligible, 31 could not be addressed due to several medical examinations in different departments of the hospital and resulting in missing time slots. Longer periods post-treatment are associated with fewer appointments for follow-ups and more medical examinations take place in 1 day. This does not necessarily mean that the children who could not be included in the study due to missing time slots are medically more complex. Ten participants refused participation. Thus, the sample included 40 participants.

Prior to addressing the participants, all eligible children and adolescents were screened using the electronic patient record (SAP® ERP). Patients were recruited during routine follow-up visits. The following inclusion criteria were applied: (1) children and adolescents during maintenance therapy and follow-up care of a pediatric oncological disease and (2) currently aged between 6 and 18 years. No restriction was applied regarding the period post-treatment. Exclusion criteria were: (1) medical contraindications such as fever, acute infection, orthopedic restrictions and mental retardation, (2) insufficient knowledge of the German language, and (3) absence of written informed consent. The attending physician confirmed participation for all recruited children and adolescents. Following these inclusion criteria, 81 children and adolescents were initially found eligible (Figure 1).

### Outcome Measures

#### Anamnestic and Anthropometric Data

Anamnestic and clinical data (i.e., type of cancer, treatment regime, end of therapy) was obtained from the electronic patient record (SAP® ERP). The nursing staff assessed anthropometric data (height and weight) during routine medical examination (seca 701 electronic column scale, seca 216 mechanical measuring rod). Body mass index (BMI) was calculated as a ratio of body weight (kg) per square body height (m^2^). By using the reference values of a healthy German cohort (25), BMI was converted into percentiles and classified in underweight <10th percentile, normal weight 10th−90th percentile and overweight >90th percentile (26).

#### Cardiovascular Parameters

Prior to the measurement, the participants had to rest for at least 10 min in a supine position. PWV, central blood pressure and peripheral blood pressure were assessed using the Mobil-O-Graph®, (I.E.M. GmbH, Stolberg, Germany and HMS Client-Server Version 5.1) an oscillometric, non-invasive method. Measurements were performed on the left upper arm. The cuff was inflated twice with a rest of 30 s in between. Cuff size was chosen according to the circumference of the participant's left upper arm. An ARCSolver Algorithm calculated the cSBP indirectly as well as the PWV from recorded brachial pulse. Raw data was transformed into z-scores and compared by using z-scores of a healthy reference cohort (27). PSBP and pDBP were compared to references from the national cohort of 4.529 children and adolescents (KiGGS study) (25). For the assessment of PWV and cSBP values, references from Elmenhorst et al. (27) of 1.445 healthy children and young adults were used. To evaluate the results of the parameters cSBP and PWV, the examined participants were separated into two groups (<8 years and ≥8 years). According to the age distribution of the reference values, participants <8 years were compared to height-matched reference values and participants ≥8 years were compared to age-matched references.

The measurement using the Mobil-O-Graph® was previously used several times in pediatric patients (28–31) and was also successfully applied in childhood cancer survivors (12).

#### Motor Performance

To quantify motor performance, the MOON test (MOtor performance in pediatric ONcology) was applied (32). The test assesses motor abilities, coordination, speed, flexibility and strength and consists of eight test items: *eye-hand coordination* (inserting pins), *static balance* (static stand), *upper extremity coordination* (throwing at a target), *speed* (reaction test), *muscular endurance* (sit-to-stand), *flexibility* (stand and reach), *hand grip strength* (hand-held dynamometry), and *muscular explosive strength* (medicine ball shot). The test lasts 20 min on average. Data of each item was compared to published age- and sex-matched reference values of a healthy population within an age range of 6 to 17 years (32). Data of participants older than 17 years was compared to reference values of healthy 17-year-olds, since reference values of healthy 18-year-olds are not available. Calculation of a total score is not possible within this tool. Instead, each item was analyzed individually and the percentage deviation to reference values was computed.

#### Physical Activity and Reintegration Into Sports Structures After Acute Treatment

Physical activity levels and status of integration into sports structures were assessed with a standardized, self-reporting questionnaire referring to the KiGGS study (25). The questionnaire was supplemented by several disease- and treatment-related aspects in accordance with the study of Kesting et al. (23) to investigate potential barriers regarding reintegration and participation in sports activities (e.g., barriers with respect to exemption from physical education at school or non-participation in sports clubs, sports therapy offers during treatment). The KiGGS study offers the reference values of healthy children and adolescents (*n* = 4.529) for comparison of our data.

### Data Analysis

Cardiovascular parameters were analyzed and compared to the healthy reference population with the one sample *t*-test. Motor performance was analyzed using the Wilcoxon signed-rank test in comparison to age- and sex-matched reference values. Pearson correlation was applied to calculate possible associations between motor performance and cardiovascular parameters, BMI and the period post-treatment. The Mann-Whitney-*U*-test was performed to evaluate anthracycline-mediated effects on cardiovascular health as well as differences within subgroups regarding different entities and levels of physical activity, motor performance, physical education at school, and achievement of physical activity recommendations.

Explorative two-sided statistical tests were conducted and *p* ≤ 0.05 was considered statistically significant. No adjustment for multiple comparison was conducted. Correlations coefficient (ρ) were classified according to Cohen (33).

Descriptive statistics were calculated with Microsoft Excel (version 15.39) for demographic characteristics and medical data. GraphPad Prism (version 8) was used to perform all further statistical analyses. Data analysis was performed in consultation with the Institute of Medical Informatics, Statistics and Epidemiology of the Technical University of Munich.

## Results

### Participants

Out of 81 eligible children and adolescents who met the inclusion criteria, a total of 40 participants (50% female) with various cancer entities were recruited and examined (Table 1).

**Table 1:** Anthropometric and medical characteristics of the participants (*n* = 40).

**Characteristics**	***N* (%)**	**Mean ± SD**	**Median**	**Range**
Age at diagnosis (years)	40 (100)	8.26 ± 4.32	9	0–16
Age at assessment (years)	40 (100)	11.28 ± 3.80	11.0	6–18
<8 years	10 (25)			
≥8 years	30 (75)			
Period post-diagnosis (years)	40 (100)	2.81 ± 3.17	1.6	0.2–14
Period post-treatment (years)	40 (100)	1.56 ± 1.79	1.04	0.2–10.33
<1 year	19 (48)			
1–5 years	20 (50)			
>5 years	1 (3)			
Leukemia/Lymphoma	18 (45)			
Bone tumor	2 (5)			
Brain tumor	7 (18)			
Other solid tumors^*^	13 (33)			
Body mass index^•^ (kg/m^2^)	40 (100)	17.63 ± 3.26	16.80	12.2–27.50
Underweight	9 (23)			
Normal weight	25 (63)			
Overweight	6 (15)			
Chemotherapy	27 (68)			
Anthracycline application	25 (63)			
Cumulative dose (mg/m^2^)		207 ± 81	227	92–354
Radiotherapy	13 (33)			
Chest-directed radiation	4 (10)			
Anthracycline + chest radiation	4 (10)			
Surgical tumor resection	19 (48)			
Relapse	12 (30)			

*Results are given in Mean ± SD (M, median; Range)*.

* *Other solid tumors: alveolar rhabdomyosarcoma (n = 1), carcinoid tumor of the appendix (n = 2), nephroblastoma (n = 3), focal nodular hyperplasia liver (n = 1), mature cystic teratoma ovary (n = 2), thoracic ganglioneuroma (n = 2), papillary thyroid carcinoma (n = 1), neuroblastoma (n = 2)*.

• *BMI was converted into percentiles and classified in underweight <10th percentile, normal weight 10th−90th percentile and overweight >90th percentile (26)*.

*Thirteen participants received a combination of anthracyclines (mainly the combination of Doxorubicin and Daunorubicin)*.

### Performed Assessments

For various reasons, not all tests could be realized with all participants. In 6/40 participants (15%), cardiovascular parameters could not be evaluated due to missing time slots during routine appointment and a lack of willingness to prolong the outpatient visit. Six of the 40 participants could not perform the MOON test due to medical limitations (current orthopedic restrictions, *n* = 2), examination-related limitations, i.e., a drain tube in the crook of the arm for follow-up MRT (*n* = 2) and abandonment due to lack of time (*n* = 2). The central venous device has already been explanted in all participants prior to our study.

Of the remaining 34 participants, not everyone performed every test item: Two participants could not perform the test item *speed* due to a drain tube in the crook of the arm. Four participants could not accomplish the test item *muscular explosive strength* due to orthopedic restrictions as well as examination-related limitations. Three participants did not perform the item *muscular endurance of the legs* (*n* = 1 had crutches, *n* = 1 severe muscular deficit in the legs, *n* = 1 lack of time). Two participants could not perform the test item *hand grip strength* with both hands due to lack of time (*n* = 1) and infusion needle in the crook of the arm (*n* = 2). The test item *upper extremity coordination* was measured in *n* = 13 participants because reference values are provided for children between 6 and 10 years only.

### Cardiovascular Health

**Figure 2:**
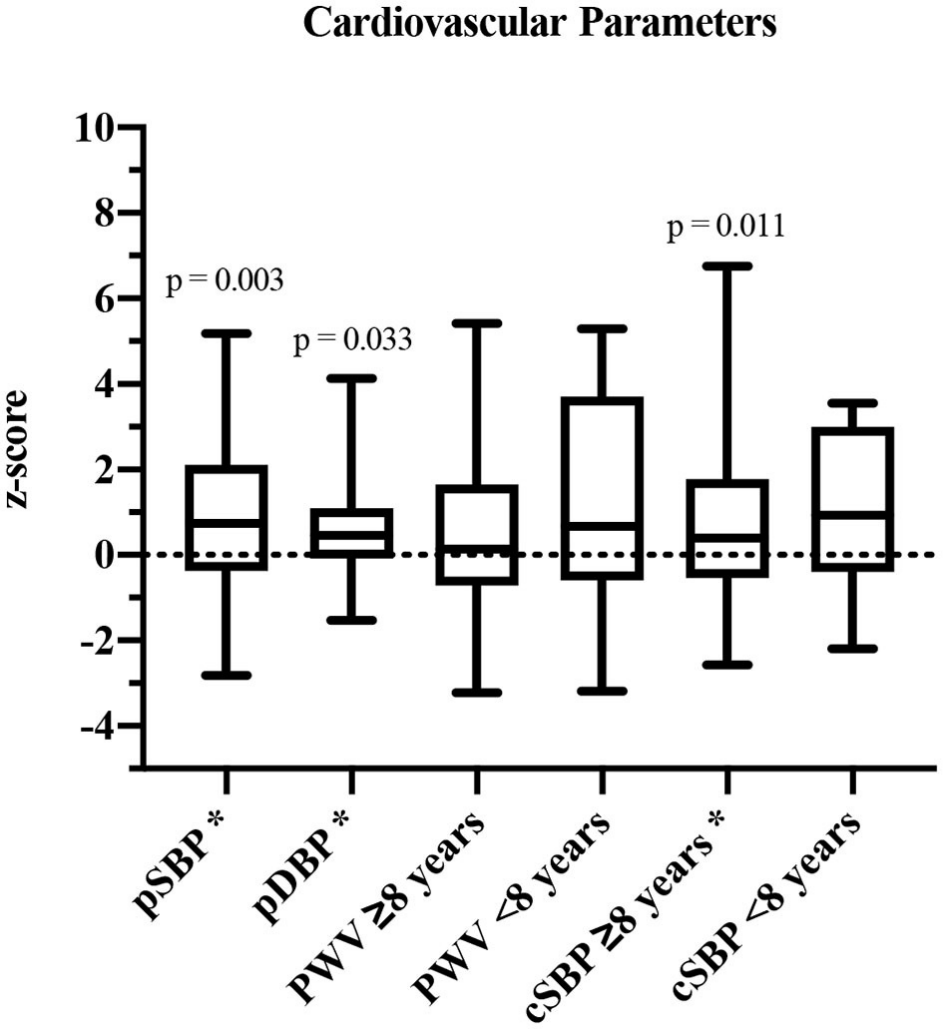
Cardiovascular parameters shown in z-scores and compared to published reference values (27). pSBP, peripheral systolic blood pressure; pDBP, peripheral diastolic blood pressure; PWV, pulse wave velocity; cSBP, central systolic blood pressure. *Significant values (*p* ≤ 0.05).

In 34/40 participants, all parameters were assessed. Based on the underlying reference values for cSBP and PWV of Elmenhorst et al. (27), the group was separated into participants aged <8 years (height-matched reference values) and ≥8 years (age-matched reference values). PSBP (z-score: 0.87 ± 1.67, *p* = 0.003), pDBP (z-score: 0.83 ± 1.94, *p* = 0.033) as well as cSBP values (≥8 years: z-score: 0.60 ± 1.29, *p* = 0.011) were significantly increased compared to reference values of healthy children and adolescents (Figure 2). PWV was elevated, but not significantly (<8 years: z-score: 1.15 ± 2.89, *p* = 0.374; ≥8 years: z-score: 0.55 ± 1.90, *p* = 0.127).

Comparison of cardiovascular parameters of 23 participants who received anthracyclines during intense therapy with a cumulative dose of 207 ± 81 mg/m^2^ and subjects who did not receive cardiotoxic agents did not show statistically significant differences.

### Motor Performance

The participants' (*n* = 34/40, 85%) motor performance was reduced in almost all motor abilities compared to the reference values of healthy children and adolescents (Table 2). Significant impairments became obvious in the following dimensions: *muscular explosive strength* (*p* < 0.001), *upper extremity coordination* (*p* = 0.032), *muscular endurance of the legs* (*p* = 0.020) and *hand grip strength* on the right hand (*p* = 0.002). The performance of *eye-hand coordination, speed, flexibility*, and *hand grip strength* on the left hand were also reduced, but not significantly. In the test item *static stand*, the study participants performed slightly better compared to the reference population. For *upper extremity coordination* (throwing at a target) only reference values from children aged 6–10 years are available. Therefore, comparison of the collected data was only possible with the same age group (*n* = 13/40, 33%). Data for older participants was not collected.

**Table 2:** Results of the MOON-test compared to reference values (*n* = 34).

**Motor ability**	**Test item**	***N***	**Mean ± SD of difference to reference values (%)**	**Media*n* (%)^#^**	***p*-value**
Eye-hand coordination	Inserting pins (time)	34	−2.12 ± 13.74	−1.08	0.599
Static balance^#^^ϕ^	Static stand (contacts)	34	−0.79 ± 7.49^#^	−1.99^#^	0.184
Speed	Reaction test (time)	32	−4.73 ± 15.48	−0.71	0.111
Upper extremity coordination^θ^	Throwing at a target (points)	13	−26.23 ± 39.16	−37.50	**0.032**
Flexibility^#^	Stand and reach (cm)	34	−1.71 ± 8.60^#^	−0.89^#^	0.278
Muscular explosive strength	Medicine ball shot (meter)	30	−20.60 ± 13.00	−22.16	**<0.001**
Muscular endurance legs	Sit-to-stand (sec)	31	−7.33 ± 24.80	−8.58	**0.020**
Hand grip strength	Hand-held dynamometry (kg)				
	Right	32	−15.41 ± 23.59	−24.65	**0.002**
	Left	33	−8.85 ± 0.17	−13.87	0.074

*All results were compared to the reference values of each single test item (32). Speed could only be measured in 32 participants, muscular explosive strength in 30 participants and hand grip strength in 32 (right) and 33 (left) participants, due to lack of time, orthopedic restrictions or drain tube in the crook of the arm*.

# *For the test items static balance and flexibility, the absolute differences were used, as the measured values would have fluctuated around zero and would have given oversized percentages*.

ϕ *Static balance was assessed counting the contacts with a foot to the ground while balancing on a rail; in this context, a negative difference to reference values represents fewer contacts and therefore better results*.

θ *Although all participants completed this test item, reference values are provided for children between 6 and 10 years only*.

*M, median; abs, indicates absolute*.

*Bold numbers indicate significant values (p ≤ 0.05)*.

Comparing motor performance of participants diagnosed with leukemia/lymphoma (*n* = 18/40, 45%) and participants diagnosed with brain tumors (*n* = 7/40, 18%) revealed some differences (Table 3). Children and adolescents treated for brain tumors performed significantly worse in *eye-hand coordination* than participants treated for leukemia/lymphoma (*p* = 0.005). Moreover, the performances in all other tested motoric dimensions of participants diagnosed with brain tumor were deteriorated compared to participants treated for leukemia/lymphomas, but not significantly.

**Table 3:** Results of the MOON-test comparing participants treated for leukemia/lymphoma and participants treated for brain tumors compared to reference values.

	**Leukemia/Lymphoma (*n* = 18/40)**			**Brain tumor (*n* = 7/40)**			
**Motor ability**	***N***	**Mean ± SD (%)**	**Median (%)^#^**	***N***	**Mean ± SD (%)**	**Median (%)^#^**	***p*-value**
Eye-hand coordination	17	1.31 ± 10.40	−1.083	6	−19.97 ± 17.32	−19.19	**0.005**
Static balance^#^^ϕ^	17	−0.55 ± 8.35^#^	−0.70	6	3.79 ± 7.567^#^	2.5^#^	0.195
Speed	16	−5.74 ± 12.32	−3.48	6	−11.60 ± 26.16	0	>0.999
Flexibility^#^	17	−1.61 ± 9.59^#^	−0.89	6	−4.75 ± 6.00^#^	−4.96^#^	0.550
Muscular explosive strength	14	−19.95 ± 16.91	−23.79	4	−20.53 ± 8.04	−19.95	0.519
Muscular endurance legs	16	−1.891 ± 30.83	−4.450	5	−13.35 ± 9.80	−8.89	0.445
Hand grip strength right	16	−7.62 ± 24.21	−9.70	5	−20.08 ± 14.82	−28.95	0.398
Hand grip strength left	17	−3.28 ± 34.33	−3.35	5	−19.98 ± 35.36	−16.49	0.595

*All results were compared to the reference values of each single test item (32)*.

ϕ *Static balance was assessed counting the contacts with a foot to the ground while balancing on a rail; in this context, a negative difference to reference values represents fewer contacts and therefore better results*.

# *For the test items static balance and flexibility, the absolute differences were used as the measured values would have fluctuated around zero and would have given oversized percentages*.

*Due to insufficient number of participants, the comparison regarding the test item upper extremity coordination was disregarded (reference values only provided for children aged 6 and 10 years)*.

*M, median; abs, indicates absolute*.

*Bold numbers indicate significant values (p ≤ 0.05)*.

**Figure 3:**
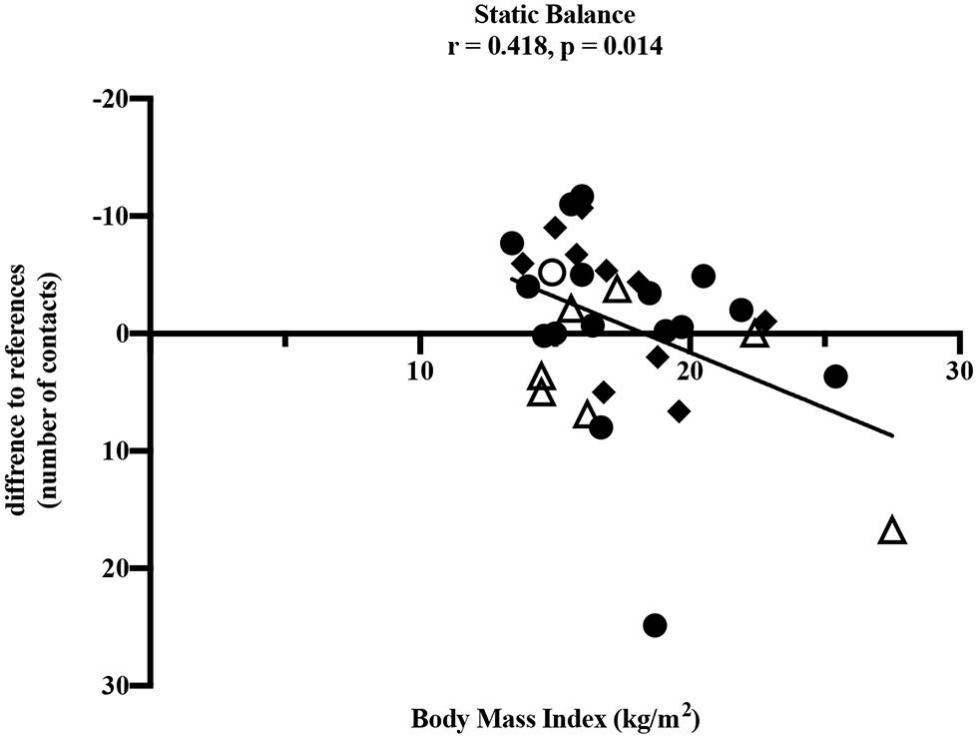
Pearson correlation between static balance and BMI (kg/m^2^). • leukemia/lymphoma, ◦ bone tumor, △ brain tumor, ◆ other solid tumors. Horizontal line indicates the reference values (25). The absolute difference from reference values is given (number of contacts to the ground). A negative difference means fewer ground contacts and therefore better performance. Thirty four participants performed the test.

To determine influencing factors on motor performance, the correlation between motor performance results and BMI as well as the period post-treatment was performed. With increasing BMI, values of *static balance* deteriorated significantly (ρ = 0.418, *p* = 0.014) which corresponds to a moderate to high correlation (Figure 3). A negative difference to reference values means fewer contacts to the ground and therefore better performance in static balance. Furthermore, some non-significant correlations were found. Deteriorated *eye-hand coordination* (ρ = −0.257, *p* = 0.143) and *flexibility* (ρ = −0.117, *p* = 0.512) were also associated with a higher BMI. However, superior values in *upper extremity coordination* (ρ = 0.317, *p* = 0.315), *muscular explosive strength* (ρ = 0.139, *p* = 0.474), *hand grip strength* (right: ρ = 0.170, *p* = 0.359; left: ρ = 0.306, *p* = 0.089) and *muscle endurance of the legs* (ρ = 0.170, *p* = 0.362) were associated with increased BMI. The test item *speed* showed no association with BMI (ρ = 0.029, *p* = 0877).

**Figure 4:**
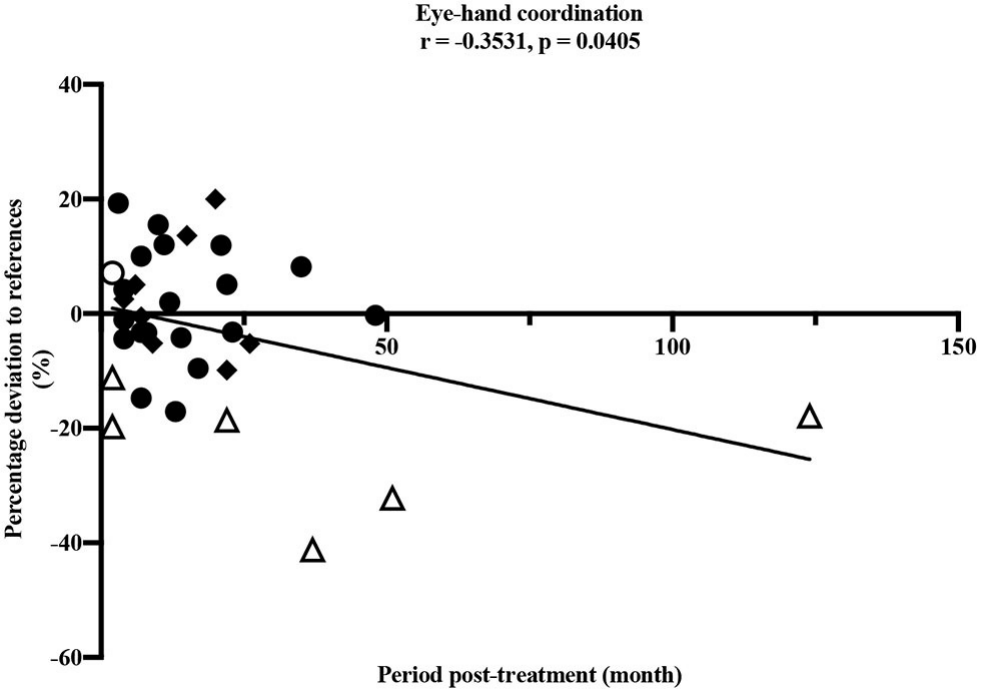
Pearson correlation between eye-hand coordination and period post-treatment (months). • leukemia/lymphoma, ◦ bone tumor, △ brain tumor, ◆ other solid tumors. Horizontal line indicates the reference values (25). Thirty four participants performed the test item.

A longer period post-treatment was significantly associated with decreased *eye-hand coordination* (ρ = −0.353, *p* = 0.041), corresponding to a moderate correlation (33) (Figure 4). Especially participants treated for a brain tumor with a longer period post-treatment showed deteriorated values in *eye-hand coordination*. Speed performance was deteriorated in participants with longer post-treatment period (ρ = −0.329, *p* = 0.066).

### Physical Activity and Reintegration Into Sports Structures

According to the self-reported questionnaire, 36% (*n* = 13/36) did not participate in physical education at school to full extend: 28% (*n* = 10/36) were not admitted to school sports activities and 8% (*n* = 3/36) were partly excluded (Table 4.1). Neither of the two participants treated for bone tumor was taking part in physical education at school (*n* = 2/36, 6%), whereas children with other tumors participated to a notably higher rate. Treatment-related muscular deficits (*n* = 2/36, 6%) and osteonecrosis (*n* = 3, 8%) were the most common reasons for participants not taking part in physical education at school.

**Table 4.1:** Participation in physical education at school subdivided into entities (*n* = 36).

	**Participation** ***N* (%)**	**Partial exemption** ***N* (%)**	**Full exemption** ***N* (%)**
Entire group (*n* = 36)	23 (64%)	3 (8%)	10 (28%)
Leukemia/Lymphoma (*n* = 16)	9 (56%)	1 (6%)	6 (38%)
Bone tumor (*n* = 2)	0 (0%)	0 (0%)	2 (100%)
Brain tumor (*n* = 6)	3 (50%)	2 (33%)	1 (17%)
Other solid tumors (*n* = 12)	11 (92%)	0 (0%)	1 (8%)

*Abs. N, number. Four out of 40 children were still attending kindergarten and could not answer the question regarding participation in physical education at school*.

Only 17% (*n* = 7/40) reported moderate-to-vigorous physical activity for 60 min daily as generally recommended by the WHO for healthy children and adolescents (Table 4.2). This percentage is comparable to the achievements in the healthy reference population (15%, *n* = 4.529).

**Table 4.2:** Physical activity and engagement in sports club and recreational sports.

	**Entire group**	Reference population^*^
	**(*n* = 40)**	**(*n* = 4.529)**
Physical activity guidelines	17%	15%
WHO (60 min/day)		
Daily physical activity		
Daily walking distance <1 km	10%	14%
Daily walking distance 1–5 km	80%	76%
Daily walking distance >5 km	10%	10%
Sports club activity (currently)	50%	58%
Former membership	27%	19%
Recreational sports activity	89%	61%

*Abs. N, number*.

* *Reference values derived from the national cohort of healthy children and adolescents in the KiGGS study (German Health Interviews and Examination Survey for Children and Adolescents) (25)*.

Every second participant questioned was an active member in a sports club, whereas 27% (*n* = 11/40) did not return to a sports club following cancer treatment. Almost one-third, 23% (*n* = 9/40), has never been a sports club member. Reasons for not engaging in sports club activities of 20 participants were: no interest/fun (40%, *n* = 8/20), physical weakness (*n* = 4/20, 20%), no time (*n* = 3/20, 15 %), anxiety (15%, *n* = 3/20), and physician-based prohibition due to clear medical reasons (10%, *n* = 2/20). Nearly all participants, *n* = 34/36 (98%), were active in recreational sports.

Further analyses pointed toward differences in physical activity and sports club participation, especially between participants with brain tumors and leukemia/lymphomas. The number of participants in recreational sports was reported high in both groups: leukemia/lymphoma (88%) and brain tumor (100%). In contrast, a difference was found in sports club activity. Sixty-six percent of leukemia/lymphoma patients were members of a sports club, whereas only 28% of participants with a brain tumor were active in a sports club. On the other hand, almost half (43%) of the children treated for brain tumor and 23% of the children treated for leukemia/lymphoma were former members.

Concerning possible correlations between motor abilities and physical education at school, participation in sports clubs or recreational sports (defined as active/inactive), as well as meeting the physical activity recommendations no significant associations could be determined. Likewise, the comparison of motor abilities of participants receiving sports therapy during treatment did not show any correlation. Almost half of the participants (45%, *n* = 18/40) took part in a sports therapy programme during treatment, which was mainly offered as care and varied greatly in terms of training interventions without any standardization.

## Discussion

The results of our study clearly present evidence for deteriorated cardiovascular function in children and adolescents shortly after cessation of cancer treatment. Increased pSBP and increased pDBP are risk factors for cardiovascular diseases, regarding guidelines for arterial hypertension (34). Potential cardiovascular consequences such as stroke, sudden death, heart failure and peripheral artery disease due to elevated blood pressure values are described in the aforementioned guidelines as well as in the literature (34, 35). Accordingly, childhood cancer survivors with elevated blood pressure are at risk to experience such cardiovascular late effects. Regarding 10-years survivors of childhood cancer, a higher prevalence of hypertension is assumed (36) and cardiovascular disease-related deaths are eight times more likely in childhood cancer survivors compared to the general population (37). Our findings support previous study results, which depict complications such as increased blood pressure, prehypertension and hypertension in children and adolescents treated for cancer (12, 38).

This study aimed at investigating specific parameters that could serve as early predictors for potential damage to the cardiovascular system. Recent evidence of cSBP, as a suitable parameter to determine the elasticity of blood vessels, suggests that cSBP is more closely related to cardiovascular events in the future than brachial blood pressure (39, 40). Increased cSBP in participants in our study may result from early changes in arterial wall stiffness. As a further parameter to detect early impairments in elasticity of the vascular system, PWV was investigated. In contrast to prior studies (7, 8), no decisive change was observed in PWV. While anthracyclines have been found to result in cytotoxic and cardiotoxic effects, these consequences have been described as a clear influence on arterial stiffness and impaired endothelial function (41, 42). In our study, we did not find any associations between increased blood pressure or PWV and anthracycline-containing chemotherapy in more than half of the participants with a cumulative dose of 207 ± 81 mg/m^2^ (range 92–354 mg/m^2^). Potentially, these consequences found in previous studies were not yet shown in our study cohort due to a shorter period post-treatment compared to other studies (41, 42). Although cSBP was increased as a sign for changes in arterial wall stiffness, consequences regarding clear outcomes might develop later and in older subjects (8) and probably more sensitive methods need to be applied.

Although the measuring methods and instruments as well as their manufacturers differ from previous studies to this one, a comparison of the results is drawn in the following. Measurements with SphygmoCor (AtCor Medical, West Ryde, Australia) showed significantly higher values of PWV in participants >18 years of age (8). However, no indication for higher PWV in participants receiving anthracyclines was found (8). Another recent study (11) examined PWV using Skidmore Medical Limited (Bristol, United Kingdom; Version 4). Their results show that PWV was elevated in 6% of children and adolescent treated for cancer after hematopoietic cell transplantation. The authors concluded that a larger waist circumference and the time of transplantation prior to the age of twelve years was associated with increased PWV (11). An ultrasound-based study showed increased arterial stiffness of the carotid artery compared to a control group in survivors of leukemia >5 years after cancer diagnosis (43).

Nevertheless, one recent study using the Mobil-O-Graph® as well, described results similar to our findings (12): The study examined n = 92 children and adolescents treated for cancer, aged 12.5 ± 4.2 years and 3.6 ± 2.8 post-diagnosis. Results show an increase in pSBP, but no significant changes in cSBP or PWV were found, independent of receiving anthracyclines. Accordingly, while our findings lend additional support to an increase in pSBP, and no significant changes in PWV, they do illustrate a significant elevation of cSBP, independent of receiving anthracyclines.

Our experience in performing the measurement with the Mobil-O-Graph® is consistently positive. Considering the compliance of the measurement with acceptance from all participants in our study was captured. The duration of the measurement was clearly accepted and did not raise any problems. Furthermore, the instrument was used with adolescents as well as very young children without encountering any difficulties.

Discrepancies of the cardiovascular outcomes in different studies and the use of diverse measurement methods support the need for further research to gain knowledge and additional insights to finally standardize the methods.

Considering the period of time post-diagnosis and post-treatment in various studies, differences in the results are discernible. In our study, participants were screened after a shorter period post-diagnosis (2.81 ± 3.17 years) and post-treatment (1.56 ± 1.79 years) compared to previous ones and showed elevated values of PWV. On the contrary, another study investigated elevated blood pressure levels, but no statistically significant variation for PWV in pediatric cancer patients compared to healthy children and adolescents was found (12). Therefore, longitudinal studies are necessary to better quantify potential temporal damages following cancer-related treatment and, in particular, changes in PWV to examine any association with the period of time post-treatment.

Our study demonstrated impairments in children and adolescents after anti-cancer treatment in nearly all dimensions of motor performance compared to healthy children and adolescents. The only exception was the test item *static stand*. In this test, participants performed better compared to the healthy reference values. It should be noted that this result would be even more distinct without the two outliers shown in Figure 3. One participant after treatment for leukemia and one participant after treatment for a brain tumor showed values remarkably high and therefore many contacts to the ground compared to all others and the reference values. However, the small sample of children treated for a brain tumor participating in our study showed clearly reduced static balance overall (Figure 3) and these impairments are well-known in this group of patients (18). Furthermore, the test item examining eye-hand coordination (inserting pins) revealed low performance in participants treated for brain tumors compared to all others and to the reference values. According to Figure 4, this seems to persist for months after cessation of treatment. However, these results should not be over interpreted due to the small number of participants and the three outliers. These three participants treated for a brain tumor with the longest periods post-treatment performed distinctly worse, but for useful results and clear interpretations a larger number of participants with long periods post-treatment needs to be examined. Measured impairments could be related to a very low level of physical activity, decreased strength and overall coordination. Thus, a lack of motoric development in these participants already begins during acute treatment (13, 14). In addition, the intense treatment with severe surgery that is required often in this group of patients should be considered. With regard to the medical treatment brain tumor patients usually receive, radiation therapy should be addressed. A total of *n* = 5/7 participants were exposed to radiation therapy. This might be an additional reason for impairments compared to other patients and healthy children. Furthermore, negative outcomes regarding motor performance due to additional radiation therapy in young brain tumor patients has been shown by Ottensmeier et al. (44). Cranial radiation therapy can be part of the treatment regime for leukemia patients as well and, therefore, could show an impact on motor skills in this group. In our study, only three out of 18 participants treated for leukemia received cranial radiation therapy. Due to this small number we refrained from any interpretations.

Our study showed impairments especially in the motoric dimensions of strength and coordination in the whole sample. Similar results were found by another study investigating pediatric cancer patients at the end of acute treatment, also using the MOON test (20). The motor abilities strength and coordination are essential to perform activities of daily living. Therefore, it seems to be of particular importance to train strength and coordination under supervised conditions already during and after treatment to counteract the loss of strength and coordination in survivors of childhood cancer. Our study did not show a clear difference in motor performance comparing participants who took part in a sports program during treatment and those, who did not (45 vs. 55%). The sports therapy program was implemented in June 2016 and offered twice a week in the beginning. Content and duration of interventions were not standardized and most of the participants took part in the care program and not in a study. Therefore, no conclusion can be drawn regarding effectiveness of our sports therapy program in the beginning, because that was not the aim of this screening. Until now, clear training recommendations and concepts for specific strength and coordination training for children and adolescents with cancer are still missing. Those instructions need to be developed and disseminated considering individuality and treatment. This would enable a more focused approach to the problem of deteriorated strength and coordination during and after therapy. *Upper extremity coordination* (throwing at a target) was tested in 13 participants aged from 6 to 10 years and found to be significantly deteriorated in our study. However, considering the small sample size studies with larger numbers of participants are needed on this motor dimension. According to subjective appraisal, participants did not have enough power to hit the target from the marked line. This assumption is reflected in the deteriorated values of *muscular explosive strength* and *hand grip strength*.

Regarding coherences between static balance and BMI as well as the period post-treatment, both variables appeared to influence single dimensions of motor performance. Supporting the results of a recent study with patients treated for bone cancer (21), our study showed that higher BMI was associated with poorer performance in *static balance*, and a longer period post-treatment was associated with deteriorated eye-hand coordination. Functional limitations with increasing BMI were shown in healthy children and adolescents as well (45). Eye-hand coordination is necessary for manual writing and handicraft work and most relevant for reintegration into school life as well as daily activities. Motoric skills like eye-hand coordination as well as flexibility should also be trained slightly during cancer treatment to counteract possible restrictions. With simple, targeted exercise interventions like throwing balls or plain strength exercises, motor skills of children and adolescents treated for cancer could continuously be trained during treatment. Exercises during intensive treatment have already been shown feasible (46–48) and well-received (49) in pediatric cancer patients. These findings correspond to our own observation since the beginning of our sports program.

Contrary to expectations, the findings of our study regarding WHO recommendations for physical activity in participants were comparable with reference values of healthy children and adolescents. But it is worth mentioning that only a low percentage of healthy peers achieve those recommendations according to the results of the KiGGS study due to a shift from usual daily physical activity in younger children to predominantly recreational sports activities in adolescents that are not performed every day (25). Every second participant was active in a sports club. Surprisingly, more participants than healthy children and adolescents were former sports club members. This result points to the fact that many children and adolescents treated for cancer do not return to a sports club early after treatment. In contrast, a higher number (nearly 90%) of participants compared to references (barley two-thirds), were active in recreational sports. The high participation rate in recreational, non-structured sports as well as a low number of currently active members of sports clubs could be referred on one hand to a short period-post treatment and a resulting lack of social integration. On the other hand, an individual training in this period might be even more useful and the level of intensity in a training in larger groups cannot be achieved yet. Further investigations regarding the main reasons are necessary.

Most children and adolescents in this study participated in physical education at school after intensive treatment and only few were exempted partly or to full extend. All participants treated for a bone tumor were exempted to the full extent and these findings agree with the results of Kesting et al. (23). Due to the small number of patients treated for bone tumors (*n* = 2) in our study, no further conclusions can be drawn. Nevertheless, future studies, should examine this entity more closely with regard to participation in physical activities/education in school, in order to define and decrease possible barriers. Participants treated for a brain tumor had a low participation in physical education at school. This result is similar to a prior study as well, where the main reason for full exemption in all entities was medical advice against sports participation by the physician (23). In contrast, this reason was mentioned only once in our study. Another study investigating patients with acute lymphoblastic leukemia almost 5 years following treatment showed that nearly all children and adolescents are participating in physical education at school. Barriers of non-participation included exhaustion or fear of injury (50). The lower participation rate in physical education at school in our study compared to the high participation rate in the previously described study is probably shown due to a shorter period post-treatment.

All in all, it should be noted, that shortly after treatment, the return to any kind of sports may not be considered as priority one for all patients and their families. Especially, during return to school, physical education might not be as important for the child's school career, but could help to reintegrate and socialize with its friends and peers.

With respect to existing barriers, the most frequently mentioned reason for full exemption from physical education at school in our study was osteonecrosis. This demonstrates that non-participation in physical education at school is not only dependent on judgement of medical or school staff, but on intra-individual reasons as well. To reduce these barriers in the future, individual and specific advice from educated trainers and therapists is needed on a regular basis and especially at the end of acute treatment and as a support during the return to normality.

Lastly, participants' motor performance was deteriorated maybe due to non-participation in physical education at school and no sufficient other type of physical activity to improve motor skills. However, these findings were not statistically significant.

To counteract impaired motor performance and deteriorated cardiovascular values, exercise and sports therapy during and after treatment should be an integral part of every department for pediatric oncology as suggested in recent reviews (46, 51). In turn, support and advice can enable children and adolescents to increase their physical activity, which might help to facilitate reintegration into sports structures and social structures after cessation of treatment.

Training during and after treatment should preferably be supervised and controlled. Findings from a recent study (52) investigating exercise in patients undergoing anti-cancer treatment, further support the promotion of exercise during and after treatment to gain physiological and functional benefits, as well as to improve quality of life. Another study published practical applications for the use of exercise for pediatric cancer patients and outlined guidelines for incorporating physical activity and exercise into daily practice in hospitals (53) that are in accordance with our own experiences in our program.

To achieve improvements in motor abilities and to counteract their decline, it is particularly important for children to remain active beyond medical treatment. This will facilitate reintegration into physical education at school, sports structures, as well as social structures.

In addition, it seems to be useful to offer professional support and advice for trainers in sports clubs on a larger scale. This enables trainers to work and qualify on disease-specific peculiarities and resilience. Therefore, better integration of formerly cancer-treated children and adolescents into sports clubs might be possible. Moreover, there should also be more training opportunities for sports teachers on how to differentiate the strain level of chronically ill children during physical education at school.

Nevertheless, trends were detected: participants who were not involved in physical education at school, who were engaging in neither sports club activities nor recreational sports, who did not meet the WHO physical activity recommendations and who did not receive sports therapy during treatment performed worse in almost all motoric abilities.

## Limitations

This study shows some limitations that are quite common and known in research within this specific sample. The group of examined participants is very heterogeneous regarding age, variety of entities, applied treatment regimens, and the range of physical impairments. Subgroup analyses point out specifics regarding different entities and show problems in groups that are usually understudied (e.g., brain tumor patients). The disadvantages of self-reporting tools like the questionnaire assessing physical activity are known (54). Recall-bias and social desirability might have distorted data, but assessment of several aspects (e.g., status of integration in sport structures and barriers) is only possible using self-reporting. Application of objective tools for cardiovascular parameters and motor performance provided reliable data. However, all measurements were performed only once. Repeated measurements could increase reliability. Recruitment was a challenge in this study. Of 81 children and adolescents eligible, 31 could not be addressed due to several medical examinations in different departments within follow-up care on 1 day and therefore only very short time slots. Most of them have appointments every 3 months or less frequent. Longer periods post-treatment are associated with fewer appointments for follow-ups. Even though this does not necessarily mean that these children are medically more complex, data of these children might have influenced the results and this aspect needs to be considered as a limitation of our study. In addition, more participants treated for a bone tumor should be studied regarding their physical impairments. However, we were able to show subjective and objective data of participants treated for different types of cancer and with various problems and needs.

## Conclusions

This study focused on cardiovascular and motor impairments of children and adolescents during maintenance and follow-up care. The analysis of their status of integration into sports structures as well as participation in physical education at school enables identification and definition of potential barriers and provides insights on how to best development of effective and helpful strategies. Affected children and adolescents should receive early support in handling their physical limitations, already during and following treatment. Early motor deficits should be revealed and reduced by applying targeted sports interventions. Implementation of sports therapy during and shortly after treatment could reduce arising therapy-related late effects, such as cardiovascular diseases. Presumably, these interventions targeted at reducing cardiovascular damage and implemented by sports therapists should be (1) initiated prior to the application of cardiotoxic agents, (2) a holistic approach including a high amount of endurance training, and (3) performed under cardiological monitoring for safety reasons and dose finding of sports interventions. Generally, children and adolescents with cancer as well as their parents should be supported and advised in every phase of treatment. Moreover, they should be encouraged to be physically active and to develop a long-term active lifestyle.

Therefore, sports programs should be included in pediatric oncology team efforts as a meaningful, cost-effective preventive approach in terms of late effects associated with physical inactivity.

## Data Availability Statement

The datasets generated for this study are available on request to the corresponding author.

## Ethics Statement

The studies involving human participants were reviewed and approved by The Ethics Committee of the School of Medicine of the Technical University of Munich. Written informed consent to participate in this study was provided by the participants' legal guardian/next of kin. Written informed consent was obtained from the minor(s)' legal guardian/next of kin for the publication of any potentially identifiable images or data included in this article.

## Author Contributions

SK and DG were responsible for conception and design of the study and the coordination of data collection. IL supervised the medical support and gave important input for drafting and revising the manuscript. TK was responsible for examination and collecting data, analyzing, and processing data. CP gave important input for the concept. RO-F gave important input for drafting and revising the manuscript. TK and SK wrote the manuscript with input from all authors, who read, and approved the final version of the manuscript.

## Conflict of Interest

The authors declare that the research was conducted in the absence of any commercial or financial relationships that could be construed as a potential conflict of interest.

## Abbreviations

PWV: Pulse Wave Velocity
WHO: World Health Organization
cSBP: central systolic blood pressure
pSBP: peripheral systolic blood pressure
pDBP: peripheral diastolic blood pressure
BMI: Body mass index
MOON: Motor performance in pediatric oncology
KiGGS: German Health Interviews and Examination Survey for Children and Adolescents.
